# Online mental health training program for male-dominated organisations: a pre-post pilot study assessing feasibility, usability, and preliminary effectiveness

**DOI:** 10.1007/s00420-023-01961-0

**Published:** 2023-02-17

**Authors:** Elizabeth Stratton, Michael J. Player, Nick Glozier

**Affiliations:** 1 Central Clinical School, Faculty of Medicine and Health, University of Sydney, Office 8, Lvl 5, Professor Marie Bashir Building, Missenden Road, Camperdown, NSW 2006 Australia; 2 ARC Centre of Excellence for Children and Families Over the Life Course, University of Sydney, Office 8, Lvl 5, Professor Marie Bashir Building, Missenden Road, Camperdown, NSW 2006 Australia

**Keywords:** Mental health, Male-dominated, Stress management, Digital

## Abstract

**Purpose:**

The emergence of digital health interventions for mental ill-health in the workplace is expansive. Digital interventions delivered in male-dominated settings are less so. This pilot study aimed to assess the usability, feasibility, acceptability, and preliminary effects of an online intervention in a male-dominated organization. We focus on male-dominated as mental ill-health is frequently unrecognized and underdiagnosed among males.

**Methods:**

Unwind, a 7-week internet-based program with stress-management components, was tested in a pre-post pilot study. Unwind gets users to identify and understand their stress triggers and assists them to develop adaptive ways to manage these and their stress. Participants were Australian adults employed in a mining company. Follow-up assessment occurred 8 weeks after baseline. The primary outcome measure was change in stress symptoms, with secondary outcomes; change in depression, anxiety, insomnia, well-being, and alcohol use. User feedback and program data were analyzed to assess usability, engagement, and intervention adherence.

**Results:**

Eligible participants *n* = 87 showed significant reductions in stress (*g* = 0.46, *p* < 0.001), depression (*g* = 0.47, *p* < 0.001), anxiety (*g* = 0.50, *p* < 0.001), insomnia (*g* = 0.44, *p* < 0.001), and well-being (*g* = 0.32, *p* = 0.004) post-intervention. Significant improvements were observed in both well and unwell (mental ill-health) and male and female participants. There was no gender effect on outcomes. A dose–response was observed as the number of modules used was related positively to improvement in anxiety (*F*_1,86_ = 5.735, *p* = 0.019; *R*^2^ = 0.25). Overall users rated Unwind as useful and engaging.

**Conclusion:**

This study presents evidence base that Unwind is a feasible and acceptable approach to reducing employees’ mental health-related symptoms in typically difficult-to-reach male-dominated industries. Unwind is feasible for larger scale delivery within male-dominated industries.

## Introduction

Mental ill-health is the leading cause of sickness absence and workplace incapacity (Murray et al. 2012). Common mental disorders account for the majority of mental ill-health seen in the workforce (Harvey et al. 2017). Mental health conditions are associated with high levels of absenteeism and reduced productivity (Harvey et al. 2011) resulting in significant costs for the individual and the organization. Common mental disorders can affect an individual’s cognitive, emotional, and social functioning (Harvey et al. 2011). Conversely, when provided with a supportive environment work can be beneficial to an individual’s overall well-being, and provide a sense of purpose and acceptance, potentially playing a critical role in the improvement of mental health symptoms (Fossey and Harvey 2010).

To address mental ill-health in the workplace, there is evidence of the effectiveness of individual-level interventions (Joyce et al. 2015). Further organizational approaches such as anti-stigma campaigns have been shown to improve mental health literacy (Hanisch et al. 2016). However, not all industries are the same, for instance, longitudinal investigations showed that those working in male-dominated industries (over 75% of the workforce are male) tend to experience higher levels of job demands and job insecurity (Milner et al. 2018).

Focus has been placed on male-dominated industries as mental health conditions are frequently unrecognized and often go undiagnosed among men (Addis and Hoffman 2017). In most OECD countries, men of working have much higher suicide rates than women (Pirkis et al. 2022). Certain aspects of “masculinity norms” and stigma lead to a reluctance in acknowledging, seek help for, and use health services for mental health problems (Addis and Hoffman 2017; Yousaf et al. 2015). This is particularly damaging in male-dominated industries as stigmatizing views can manifest into negative behaviors such as bullying and discrimination which can have detrimental effects as discrimination has been linked with depression bio-directionally (Stratton et al. 2020). Higher rates of suicide in males in such industries compared to males working in female-dominated industries (Milner and King 2019) has produced industry-specific initiatives such as mates in construction and mates in mining.

For these reasons, there is a need to implement strategies to address men’s mental health help-seeking, particularly in male-dominated industries. One potential approach is the use of eHealth interventions, as they provide workplaces with the ability to deliver easy, cost-effective tools universally to the workplace (Harrison et al. 2011). Importantly, for male-dominated industries eHealth interventions are delivered anonymously, allowing employees to seek help without concern of stigma. A meta-analytic review showed that eHealth interventions are effective at addressing employees’ high-stress and common mental health concerns, particularly stress-management interventions (Stratton et al. 2022). However, evidence is lacking specifically for the effectiveness of eHealth in male-dominated industries. A meta-analysis of RCTs evaluating workplace eHealth show that a majority (64%) of employees that use such workplace interventions are female (Stratton et al. 2022), suggesting that developers should be focused on designing eHealth interventions that target males to potentially increase uptake and utilization in male-dominated industries.

Therefore, in this pilot study, we sought to examine the feasibility, usability, acceptability, and potential preliminary effects of an eHealth intervention, Unwind, focusing on health and well-being delivered in a male-dominated industry within an Australian mining company.

## Methods

### Participants and setting

This study was conducted among the employees of an Australian mining organization in six mining sites. Due to the nature of mining, the sites were based in remote locations, often several kilometers from the nearest road with limited facilities. Employees were a mix of fly-in fly-out, drive-in drive-out, or home-based. Fly-in fly-out is often a method used in mining due to remote locations for employing people by flying them temporarily to the work site instead of relocating employees and their families permanently. Inclusion criteria were (1) aged at least 18 years, (2) current employee, (3) sufficiently fluent in English, and (4) having Internet access for the eight-week duration of the study.

### Recruitment process

An email invitation was sent out to all employees who had participated in a prior in-house workplace health and well-being survey. The email invitation contained a link to the participant information statement and consent form. Once participants had provided informed consent, they completed the baseline assessments and were given access to Unwind, a stress-reduction program designed for this male-dominated and remote context. Recruitment, consent, and data collection were all conducted online. The study was conducted between February and April 2017. Ethics approval was granted by the University of Ethics Committee (ref number: 2016/741).

### Intervention

**Fig. 1:**
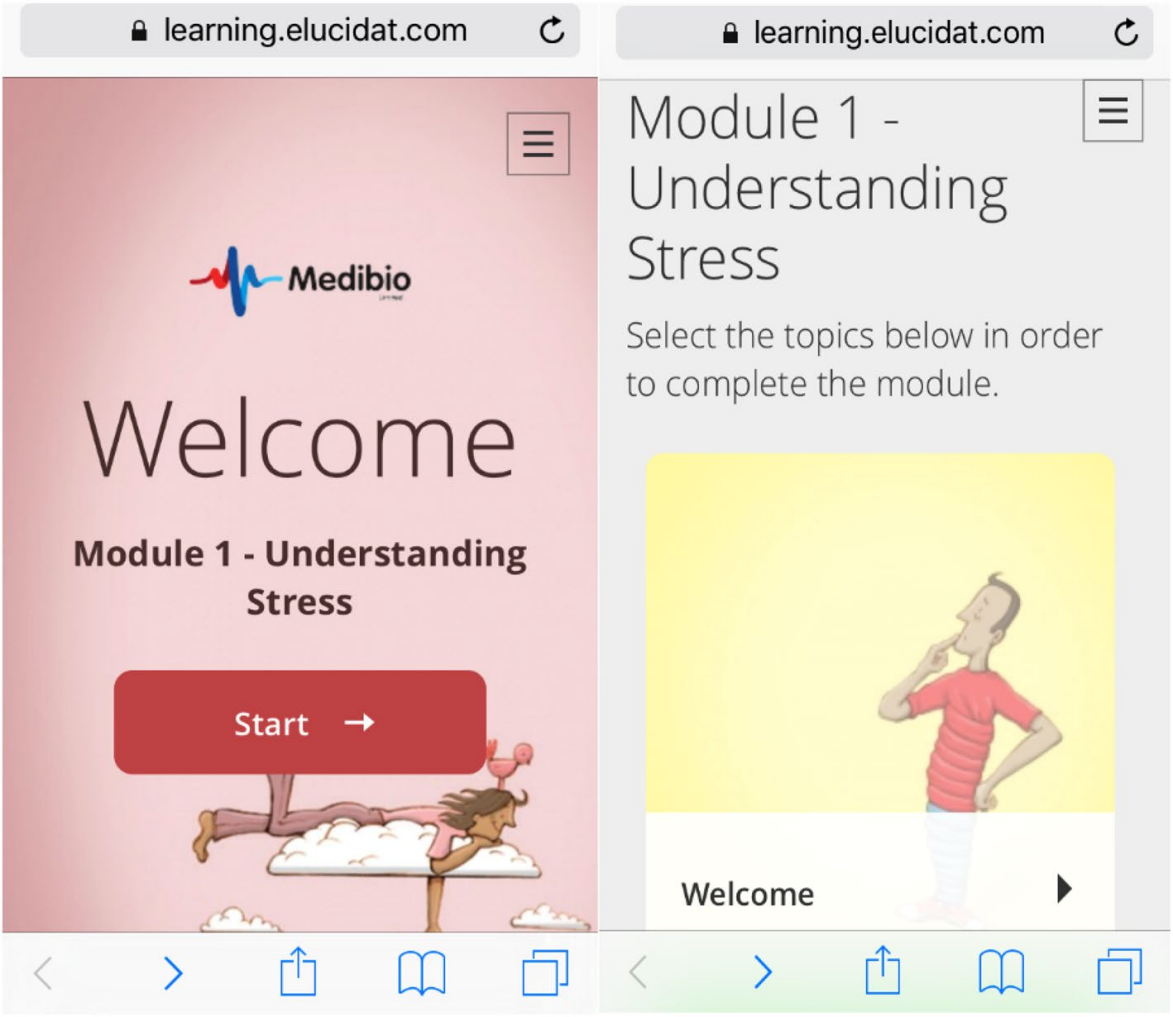
Screenshots of unwind stress-reduction program

‘Unwind’ (see example Fig. 1), is an internet-based stress-reduction program delivered via a web browser and optimized for smartphone viewing. The 7-week program delivers one module per week. Users were unable to move to the next module without completing the previous weeks module. ‘Unwind’ gets users to identify and understand their stress triggers and assists them to develop adaptive ways to manage these and their stress. It provides evidence-supported tools for users to manage symptoms of arousal via breathing techniques, progressive muscle relaxation, and mindfulness. Users identify important elements for their health such as establishing an appropriate exercise regime, improving sleep, reducing alcohol, smoking, and caffeine intake, maintaining a balanced diet, and taking time out to restore energy levels. Interactive elements include animation, audio, and video content, such as, ‘visualization’ exercises for stress reduction. The program also asks users to reflect on challenging experiences to build resilience and build positive coping options. Throughout the modules, learning and understanding were reinforced through guided questionnaires and quizzes, homework tasks, and pre-loaded feedback. The program culminates in the development of a personalized ‘Well-being Plan’ in the final module.

### Measures

Baseline measures were assessed before starting the program. Later assessments were collected either within the program or by the user being electronically directed to an online survey at the end of the program when they indicated they finished or had not completed any modules for three works.

#### Demographic and workplace

Participants self-reported demographic information including; gender and age (in years).

#### Mental ill-health and well-being

Mental ill-health symptoms were assessed via the Depression, Anxiety, and Stress Scale (DASS-21; (Lovibond and Lovibond 1995)) three self-report 7 item sub-scales assessing anxiety, depression, and stress symptoms. Higher scores indicate more mental health symptoms. It has good reliability and validity (Cronbach’s alpha 0.94, 0.87, and 0.91 for depression, anxiety, and stress sub-scales respectively, Antony et al. 1998; Ng et al. 2007), along with good construct validity (Henry and Crawford 2005). Because the DASS-21 is a short-form version of the original DASS (42 items), each 0–3 rated item is multiplied by two for comparison with the original DASS scores where the following cut-points indicate severe symptoms; depression =  > 21, anxiety =  > 15, and stress =  > 27 (Antony et al. 1998; Lovibond and Lovibond 1995).

#### Insomnia

Assessed using the 6-item Bergen Insomnia Scale (BIS) (S. Pallesen et al. 2008a, b) (Cronbach *α* = 0.62). The BIS is a widely used scale constructed to identify clinical diagnostic criteria for insomnia and validated against polysomnographic data (Stäle Pallesen et al. 2008a, b).

#### Alcohol use

Measured using the alcohol consumption questions (AUDIT-C) (*α* = 0.53), a three-item alcohol screen for hazardous drinking, a score of 3 or greater for women and 4 or greater for men indicated “at risk” drinking (Bush et al. 1998).

#### Well-being

The World Health Organisation Well-Being Index (WHO-5) (Cronbach *α* = 0.80), assesses global well-being using five items rated on a 6-point Likert scale: higher scores indicating superior well-being (Bech 2004). The WHO-5 scale has adequate validity as an outcome measure in clinical trials and has been applied successfully across a wide range of study fields (Topp et al. 2015).

#### Usage

Was determined through the objective metric of module completion.

#### Engagement

Module-specific engagement with Unwind was assessed through 7 items Likert scale questions embedded in each module, for instance, “Rate how you felt about this module: waste of time, not that useful, boring, average, somewhat useful, enjoyable, excellent”. After each module use, participants were asked to self-report their general mental health state: “select how you are feeling at the moment 1 = great, 2 = good, 3 = ok, 4 = steady, 5 = poor, 6 = rubbish, 7 = help!”.

Overall acceptability was rated “On a scale of 0–5, Please tick the best option for the following questions (a) How do you rate the program in terms of user-friendliness, and (b) What was your overall impression of the program? (Excellent, Very Good, Good, OK, Poor, Very Poor)”.

### Statistical analysis

All data were analyzed with SPSS V.26. The sample characteristics were described using appropriate mean/standard deviation, and number/proportion (%) to present continuous and categorical data respectively.

Primary analyses were undertaken on an intention-to-treat basis, including all eligible participants. Little's test of the missing completely at random mechanism was conducted to determine the quality of missingness. As this test was not significant multiple imputation approach was used for missing data. We used the multiple imputation (MI) procedure to impute missing sum scores for participants who did not complete the post-assessments. Ten single imputations of the missing values were calculated based on the valid data for all outcome measures at baseline and aggregated into a single value. MI is considered to be a conservative approach to analyzing incomplete datasets (Schafer and Graham 2002). A significance level of 0.05 (two-sided) was used for all analyses.

Analysis of potential preliminary effectiveness compared changes between baseline and post-intervention (8-weeks) scores using paired samples *t* tests. Cohen’s *d* was calculated by comparing the change in means post-intervention. According to Cohen, *d* = 0.2 can be considered small, *d* = 0.5 a moderate effect, and *d* = 0.8 a large effect (Cohen 1988). Independent sample *t* tests were used to compare the difference in change score between a) those that were unwell and well and; b) between males and females using baseline and post-intervention scores across all outcomes. Analysis of covariance (ANCOVA) was used to explore gender impacts on change scores while adjusting for baseline scores. Finally, linear regression analysis was used to explore dose-response, in this case, the dose is referred to by the number of modules used by participants.

## Results

**Fig. 2:**
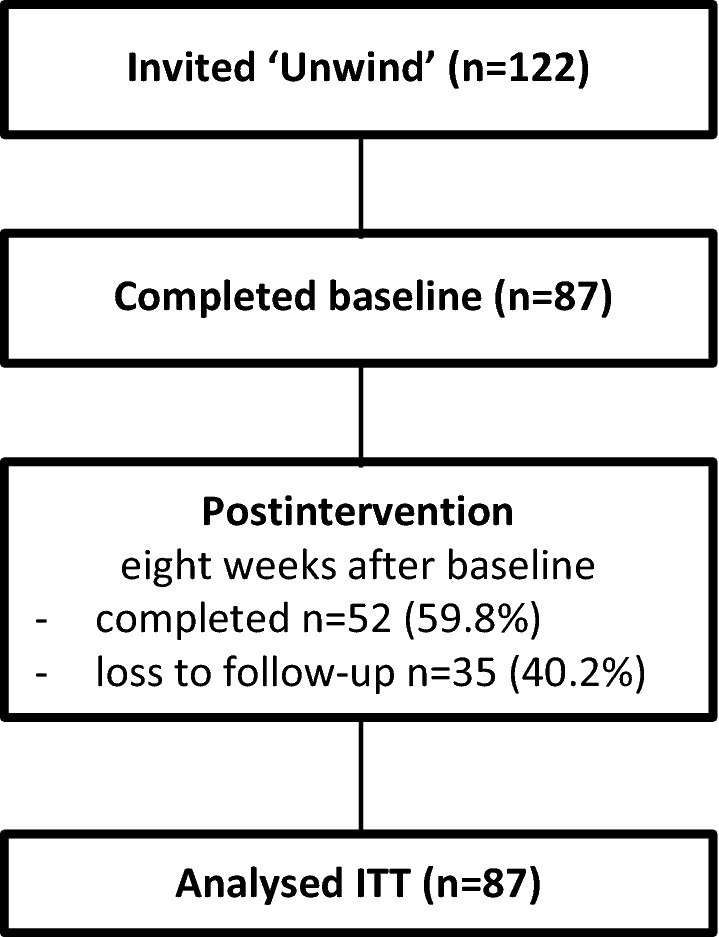
Flowchart of study participants

Eighty-seven of one hundred and twelve (78%) invited employees used the ‘Unwind’ intervention. Of these, 52 (60%) persisted with the study and provided follow-up data (Fig. 2). Only one baseline item differed between those who persisted with the study (study completers) and those lost to follow-up (Table 1). Non-completers reported poorer relationships with their boss, and men were just as likely as women to complete the study. (Table 1).

**Table 1:** Participant baseline demographics and health factors overall and comparing completers and non-completers

Variable	Unwind ITT (*n* = 87)	Unwind completers (*n* = 52) 60%	Unwind non-completers (*n* = 35) 40%	Significance test *p* value
Gender	*N* (%)	*N* (%)	*N* (%)	
Male	53 (60.92)	31 (59.62)	22 (62.86)	0.761
Female	34 (39.08)	21 (40.38)	13 (37.14)	

	M (SD)	M (SD)	M (SD)	
Age	39.06 (8.87)	38.87 (9.33)	39.27 (8.43)	0.836
Stress	17.72 (9.13)	17.26 (9.17)	18.24 (9.16)	0.619
Depression	12.11 (9.11)	12.00 (9.92)	12.24 (8.24)	0.902
Anxiety	9.82 (8.20)	8.83 (8.01)	10.93 (8.37)	0.235
Well-being	14.31 (5.11)	13.82 (5.01)	14.85 (5.24)	0.354
Insomnia	13.02 (7.50)	14.02 (7.67)	11.90 (7.17)	0.188
Risky alcohol use	4.06 (2.62)	3.96 (2.49)	4.24 (283)	0.615

### Participant characteristics

The mean age was 39.1 years (range: 23 to 58) (Table 1) and the majority were male (61%). Elevated symptoms of stress were observed in over half of this population (51.7%). In the overall sample, the average mean mental health scores in this sample show mild to moderate levels of stress (*m* = 17.7), depression (*m* = 12.1), and anxiety (*m* = 9.8) according to the DASS-42 (Lovibond & Lovibond 1995).

### Health outcomes

All measures of mental ill-health and well-being improved after the use of ‘Unwind’ (Table 2) with moderate effect sizes. However, there was no observed effect on alcohol use behaviors. The ITT analysis shows that the mean depression, anxiety, and stress scores after using ‘Unwind’ were in the ‘normal’ range in the overall sample at post-intervention use (Table 2).

**Table 2:** Mental-Ill health and Well-being outcomes

	Baseline (*n* = 87) mean (SD)	Follow-up (*n* = 87) mean (SD)	Mean diff	*t* score	*p* value	Cohen’s *d*
Stress	17.72 (9.13)	14.02 (6.72)	− 3.70	3.978	< 0.001	0.46
Depression	12.11 (9.11)	8.21 (7.21)	− 3.90	5.411	< 0.001	0.47
Anxiety	9.82 (8.20)	6.32 (5.69)	− 3.50	4.610	< 0.001	0.50
Insomnia	13.02 (7.50)	10.22 (4.79)	− 2.80	3.987	< 0.001	0.44
Well-being	14.31 (5.11)	15.72 (3.61)	1.41	2.951	0.004	0.32
Risky alcohol use	4.06 (2.62)	3.94 (2.61)	− 0.12	0.759	0.452	–

Approximately one-third of this population would be classified as unwell using DASS-42 cut points (Lovibond and Lovibond 1995), with 52% reporting high stress, 39% reporting what would be considered clinical levels of depression, 31% anxiety, and 32% insomnia. Low levels of well-being were reported in 37% of this sample. Finally, high levels of alcohol use were reported in 56% of this sample.

When considering the impact of Unwind on users that were well or unwell on each measure, we notice a clear difference. Although positive outcomes are reported for all measures in the ‘well’ group a significant reduction was only observed in depression. Whereas those that were considered ‘unwell’ show clear improvements in overall mental health and well-being outcomes (Table 3) with large effect sizes. Again, no change was observed in risky drinking practices.

**Table 3:** Outcomes in well and unwell participants

	*n*	Baseline mean (SD)	Follow-up mean (SD)	Mean diff	*t* score	*p* value	Cohen’s *d*
Well							
Stress	42	15.71 (8.67)	13.43 (6.92)	− 2.28	1.839	0.073	–
Depression	62	7.42 (5.39)	5.77 (3.59)	− 1.65	2.619	0.011	0.36
Anxiety	60	5.07 (3.78)	4.47 (3.13)	− 0.60	1.181	0.243	–
Insomnia	59	10.15 (6.36)	9.29 (4.58)	− 0.86	1.147	0.256	–
Well-being	55	17.51 (2.75)	1.06 (2.83)	− 0.45	1.068	0.290	–
Alcohol use	48	1.41 (0.91)	1.45 (1.14)	0.45	0.326	0.747	–
Unwell							
Stress	45	19.60 (9.23)	14.58 (6.56)	− 5.02	3.697	0.001	0.63
Depression	25	23.76 (5.04)	14.24 (10.02)	− 9.52	6.452	0.000	1.20
Anxiety	27	20.37 (4.71)	10.44 (7.69)	− 9.93	6.262	0.000	1.56
Insomnia	28	19.07 (5.89)	12.16 (4.71)	− 6.91	5.764	0.000	1.30
Well-being	32	8.65 (2.94)	13.44 (3.74)	4.79	5.595	0.000	1.42
Alcohol use	49	6.07 (1.38)	5.83 (1.63)	− 0.24	0.960	0.345	–

Given the focus on developing a program acceptable to male employees, we evaluated the outcomes by gender, in this case, male and female. Significant improvements were observed in both males and females. In females, symptoms relating to stress, depression, anxiety, and insomnia significantly improved (Table 4). Similarly, in males, significant improvements in stress, depression, anxiety, insomnia, and well-being were observed. Across all outcome measures, females improved more than males.

**Table 4:** Outcomes across gender

	Baseline mean (SD)	Follow-up mean (SD)	Mean diff	*t* score	*p* value	Cohen’s *d*
Males (*n* = 53)						
Stress	16.49 (9.21)	14.00 (6.55)	− 2.49	2.041	0.046	0.55
Depression	11.32 (9.04)	8.04 (6.39)	− 3.28	3.500	0.002	0.76
Anxiety	9.21 (7.07)	6.42 (5.22)	− 2.79	3.194	< 0.001	0.72
Insomnia	11.60 (7.38)	9.55 (4.66)	− 2.05	2.143	0.037	
Well-being	14.63 (5.16)	15.93 (3.50)	1.30	1.987	0.052	0.00
Alcohol use	4.45 (2.58)	4.43 (2.73)	− 0.33	0.154	0.879	0.386
Females (*n* = 34)						
Stress	19.65 (8.77)	14.06 (7.08)	− 5.59	4.014	< 0.001	0.69
Depression	13.35 (9.22)	8.47 (8.43)	− 4.88	4.331	< 0.001	0.74
Anxiety	10.76 (9.74)	6.18 (6.43)	− 4.59	3.335	0.002	0.57
Insomnia	15.23 (7.16)	11.26 (4.86)	− 3.98	4.027	< 0.001	
Well-being	13.82 (5.09)	15.48 (3.83)	1.66	2.253	0.031	0.39
Alcohol use	3.53 (2.68)	3.24 (2.32)	0.99	1.096	0.286	0.24

The only significant differences at baseline between males and females were insomnia scores, symptoms of insomnia were higher in females. Although Unwind was seemingly more beneficial for females, there were no significant differences in change scores across any health outcomes by gender (Table 5).

**Table 5:** Gender effects on health outcomes

	Mean baseline male (*n* = 53) mean (SD)	Mean baseline female (*n* = 34) mean (SD)	*p* value	Mean change male (*n* = 53) mean (SD)	Mean change female (*n* = 34) mean (SD)	Mean diff	*F* score	*p* value
Stress	16.49 (9.21)	19.65 (8.77)	0.116	− 2.49 (8.88)	− 5.59 (8.12)	3.10	0.514	0.476
Depression	11.32 (9.04)	13.35 (9.22)	0.313	− 3.28 (6.82)	− 4.88 (6.57)	1.60	0.326	0.569
Anxiety	9.21 (7.07)	10.76 (9.74)	0.391	− 2.79 (6.37)	− 4.58 (8.02)	1.79	0.586	0.446
Insomnia	11.60 (7.38)	15.23 (7.16)	0.026	− 2.06 (6.99)	− 3.97 (5.76)	1.91	0.384	0.537
Well-being	14.63 (5.16)	13.82 (5.09)	0.476	1.52 (4.94)	1.66 (4.29)	− 0.14	0.055	0.814
Alcohol Use	4.45 (2.58)	3.53 (2.68)	0.112	− 0.03 (1.19)	− 0.24 (1.00)	0.21	0.942	0.337

### Usage and engagement

#### Usage

All participants (*n* = 87) started the intervention, Module 1 was completed by 83 of the participants (95.4%), Module 2 by 61 (70.1%), Module 3 by 54 (62.1%), Module 4 by 44 (50.6%), Module 5 by 41 (47.1%), Module 6 by 36 (41.1%), and Module 7 by 32 participants (36.7%). There were no significant differences between males’ and females’ use of Unwind. The number of modules used was related positively to improvement in anxiety (*F*_1,86_ = 5.735, *p* = 0.019; *R*^2^ = 0.25) but no other measures. The average time spent engaged in the intervention was 417 min, (6.95 h).

#### Engagement

Overall, Unwind was rated as “somewhat useful” by 44%, and “enjoyable” or “excellent” by a further 36%, with fewer than one in twenty ratings as “boring” or “not that useful”.

## Discussion

This pilot study evaluated the feasibility, usability, and potential effectiveness, of a new digital stress reduction program, Unwind, in a male-dominated industry. Unwind proved to be a feasible intervention within this male-dominated organization as participants reported a reduction in stress, depression, anxiety, and insomnia symptoms and increased participant well-being, and users reported high levels of engagement. Importantly the majority of participants were male, and the men showed similar levels of response to the female users with no significant differences observed in change scores.

The success of digital mental health interventions in treating symptoms in employees is well established (Carolan et al. 2017; Phillips et al. 2019; Stratton et al. 2017). However, evidence within male-dominated and remote industries where access to health care is low, such as mining, is lacking. In this mining organization, we observed moderately strong within-group effect sizes in all mental health outcomes. Although moderate, these observed effects could be clinically meaningful on a population level considering we used a universal approach delivered to all employees, which likely included employees that had no clinical diagnosis of mental health conditions. The moderate effect sizes observed in this study show that Unwind outperforms the average impact of digital eHealth delivered to employees when compared to meta-analytic findings where small effects are reported for stress (*g* = 0.25), depression (*g* = 0.26), and anxiety (*g* = 0.26) (Stratton et al. 2022). Interestingly, we observe an even larger positive effect across mental health outcomes in those participants that met the criteria for clinical levels of mental health diagnoses. Suggesting that Unwind is a feasible intervention when delivered universally and even more so when delivered to unwell employees.

We did not see any significant gender effects across any outcome measures. However, in each outcome, a trend for greater positive impacts were observed in females. Suggesting that future versions of this intervention might consider a male focus by the use of male end-users in participatory design to understand more about the features that might impact positive change and adherence.

Addressing conditions such as depression in the workplace has been widely promoted as a priority area for interventions (Cuijpers et al. 2012), and recent evidence has shown some effect of eHealth interventions on employees’ function (Deady et al. 2020). There is less evidence of the impact of digital interventions on employees’ sleep, most coming from sleep-specific interventions (Espie et al. 2012). In this study, the improvement in insomnia symptoms was as strong as that of other symptoms. Given the impact of sleep disturbance on workplace injuries, health and safety, (Pilcher and Morris 2020) this impact on insomnia symptoms needs to be evaluated further for potential other benefits, especially in such a sample group where shift work is common.

In terms of intervention adherence, the results of this study are extremely promising with 71% of users completing at least three of seven modules and 36% completed all seven modules. This is higher than adherence rates seen in similar feasibility studies in both non- and male-dominated industries (Collins et al. 2020; Deady et al. 2018). This could be of particular importance as employees in male-dominated industries are classically non-help-seeking and more difficult to reach with treatment for mental health concerns (Addis and Hoffman 2017; Yousaf et al. 2015). One potential hypothesis for the increased inherence is within the design of the intervention. The advertisement of this study and the wording within the modules were based around “improving health and well-being” positive psychology focus words rather than focusing on asking users to treat illness (Slade 2010). Engagement time was also promising with users on average spending one hour per week on the intervention. In a clinical treatment sense, one hour per week being exposed to an intervention aimed at improving psychological health and well-being could have a considerable impact on this hard-to-reach working population. Similarly, to other mental eHealth interventions, higher program usage was positively correlated with greater improvements in certain symptoms (Donkin and Glozier 2012), suggesting that a positive dose-response.

User feedback regarding the content and usability of the intervention was generally positive. With a majority, rating the intervention content as somewhat useful or enjoyable. Further, a majority of users’ reports were positive for usability, with 67% rating the program as good and 33% rated the program as excellent in terms of user-friendliness. These findings, however, are limited to the users that completed the program, these findings do not include the participants that were lost to follow-up.

### Limitations

This study is not without limitations. First, this is a pilot uncontrolled study. A randomized controlled trial is required to confirm preliminary findings. Second, recruitment for this study was from a group already interested in improving health and well-being which may have inflated engagement and adherence. Only 35% of participants completed all seven modules. However, this is higher than the few similar studies in male-dominated industries (Collins et al. 2020; Deady et al. 2018) which showed 32% and 21% completing these programs. Finally, this study was limited to self-reported questions. Nevertheless, how people feel and perceive reality is core to the development of mental ill-health, and has, in the case of work-related factors, been shown to be more relevant than objective measures (Glozier et al. 2010) .”

## Conclusion

This study presents evidence base that eHealth interventions have the potential to be effective at reducing employees’ mental health-related symptoms in typically difficult-to-reach male-dominated industries, such as mining. The results suggest that the delivery of Unwind is feasible for larger-scale delivery within this male-dominated industry, was well-received in terms of usability and acceptability as well as showing very promising levels of adherence and engagement within a typically difficult-to-reach population of mining employees in male-dominated industries. Recommendations to explore the outcomes in a larger scale randomized controlled trial is warranted. End-users, managers, and industry experts should be engaged to further examine the efficacy in focus groups to enhance the practical application of the research prior to large-scale implementation.

## Data Availability

The deidentified datasets generated during and/or analysed during the current study are available from the corresponding author on reasonable request.
